# Epidemiology, Molecular Pathogenesis, Immuno-Pathogenesis, Immune Escape Mechanisms and Vaccine Evaluation for HPV-Associated Carcinogenesis

**DOI:** 10.3390/pathogens12121380

**Published:** 2023-11-23

**Authors:** Meenu Jain, Dhananjay Yadav, Urmila Jarouliya, Vishal Chavda, Arun Kumar Yadav, Bipin Chaurasia, Minseok Song

**Affiliations:** 1 Department of Microbiology, Viral Research and Diagnostic Laboratory, Gajra Raja Medical College, Gwalior 474009, Madhya Pradesh, India; 2 Department of Life Science, Yeungnam University, Gyeongsan 38541, Republic of Korea; dhanyadav16481@gmail.com; 3 SOS in Biochemistry, Jiwaji University, Gwalior 474011 Madhya Pradesh, India; urmi_gudia@rediffmail.com; 4 Department of Pathology, Stanford School of Medicine, Stanford University Medical Center, Palo Alto, CA 94305, USA; chavdavishal2@gmail.com; 5 Department of Microbiology, Guru Gobind Singh Medical College and Hospital, Baba Farid University of Health Sciences, Faridkot 151203, Punjab, India; arunbiochemju@gmail.com; 6 Department of Neurosurgery, Neurosurgery Clinic, Birgunj 44300, Nepal; trozexa@gmail.com

**Keywords:** HPV-infection, HPV-associated carcinogenesis, immuno-pathogenesis, immune evasion, vaccination

## Abstract

Human papillomavirus (HPV) is implicated in over 90% of cervical cancer cases, with factors like regional variability, HPV genotype, the population studied, HPV vaccination status, and anatomical sample collection location influencing the prevalence and pathology of HPV-induced cancer. HPV-16 and -18 are mainly responsible for the progression of several cancers, including cervix, anus, vagina, penis, vulva, and oropharynx. The oncogenic ability of HPV is not only sufficient for the progression of malignancy, but also for other tumor-generating steps required for the production of invasive cancer, such as coinfection with other viruses, lifestyle factors such as high parity, smoking, tobacco chewing, use of contraceptives for a long time, and immune responses such as stimulation of chronic stromal inflammation and immune deviation in the tumor microenvironment. Viral evasion from immunosurveillance also supports viral persistence, and virus-like particle-based prophylactic vaccines have been licensed, which are effective against high-risk HPV types. In addition, vaccination awareness programs and preventive strategies could help reduce the rate and incidence of HPV infection. In this review, we emphasize HPV infection and its role in cancer progression, molecular and immunopathogenesis, host immune response, immune evasion by HPV, vaccination, and preventive schemes battling HPV infection and HPV-related cancers.

## 1. Introduction

Human papillomavirus (HPV) is an infectious agent that contributes to sexually transmitted diseases (STDs) worldwide. Sexually active individuals are at a high risk of infection during their lifetime, with 80% of women being more susceptible [1]. In addition, 49% of men have been infected with some type of genital HPV [2]. HPV infection is observed in >90% of cervical cancers [3]. However, HPV is not the only virus participating in cervical cancer development; other hazardous situations such as coinfections (with herpes simplex virus type-2 and Chlamydia trachomatis), smoking, persistent use of oral contraceptives, multiparity, nutritional deficiencies, immunosuppression, and immune-associated diseases are also involved in cancer progression [4,5,6,7,8]. Host immune responses fight against HPV infection and eliminate most of the viral part (nearly 90%) within 2–3 years or remain in the dormant phase, while the remaining 10% are converted into chronic infections; however, only 1% can cause cervical cancer [9]. Appropriate innate and adaptive immune responses and efficient immune control mechanisms are required to inhibit HPV infection. However, HPV can use the evasion process to escape the immune response. Therefore, HPV can multiply in host cells during viral replication without causing cytolysis, which neither stimulates the inflammatory process nor presents viral antigenic representatives. In host cells, type-I IFNs levels are reduced by the E6 and E7 proteins of HPV16 and generate an immune tolerance stage in the absence of costimulatory factors through inflammatory cytokines [10]. In addition, its E5 protein controls the reduced expression of class 1 HLA, which further leads to the obstruction of CTL assault [10]. These immune escape phenomena may contribute to the existence and stability of HPV infection, which further addresses cancer progression. Generally, HPV infection seems to be clinically silent because it does not show any significant symptoms; however, a few lesions can be observed in genital organs that might convert into invasive cancers [11]. Persistent HPV infection and a lower host immune response collectively support instigating carcinogenesis by conversion of a low-grade squamous intraepithelial lesion (LSIL) into a high-grade squamous intraepithelial lesion (HSIL), which ultimately transforms into an invasive form of cervical carcinoma [12]. The involvement of HPV in cancer induction and progression can take many years for conversion into a carcinoma with the help of different tumor-stimulating steps, such as E6/E7 proteins that interact with cellular proteins. This ultimately transforms normal cells into cancerous cells with immortal, proliferative, and malignant characteristics [5]. Additionally, a few reports have suggested that imbalanced immunity and chronic inflammation in the tumor microenvironment (TME) may provoke precancerous cervical lesions that turn into invasive cancer [13,14,15]. In this article, we reviewed the prevalence status of HPV infection, its mechanistic role in carcinogenesis, its molecular and immunopathogenesis process, host immune response activated by HPV, its immune escape mechanism, its vaccination scheme, and preventive measures and strategies that could protect against HPV infection and associated cancers.

## 2. Epidemiology of HPV-Induced Cancers

Human Papilloma Virus (HPV) is the most common sexually transmitted viral infection disease in women and men worldwide. At some point in their lives, 85% of women and 95% of sexually active men are infected with HPV [16]. Virtually all cases of cervical cancer, most anal cancers, and a substantial percentage of noncervical malignancies, such as vaginal, penile, vulvar, and oropharyngeal cancers, are also caused by HPV. With an estimated 604,127 new cases and 341,831 deaths in 2020, cervical cancer is the second most common cancer between ages 15 and 44 years in the world [17,18]. Higher rates of infection are prevalent in India, Eastern Europe, Latin America, and sub-Saharan Africa [19]. Globally, its prevalence fluctuates significantly and is higher in developing regions. Nearly 90% of cervical cancer-related deaths occur in low- and middle-income countries (LMICs). The fractions linked to HPV infections and associated cancers vary across geographical regions and levels of economic development. Compiled studies from cytologically healthy women showed that HPV prevalence was greater in Sub-Saharan Africa (SSA) (24.0%), especially in Eastern Africa (33.6%) and Latin America [17,20]. The highest prevalence of HPV among females was observed in Asian regions, where nearly half of the Eastern Asia (China), and South Central Asia (India) (57.7 and 44.7%, respectively), were carriers. In the SSA region, 42.2% of women in Southern and 32.3% of women in Eastern Africa were HPV carriers, respectively. In nearly all European countries, HPV prevalence is minimal (<30%), as in Western Europe (3.7%). Hence, HPV infection rates were greater in the developing regions (42.2%) than in the developed regions (22.6%) [21,22].

Numerous strains of HPV (16, 18, 26, 31, 33, 35, 39, 45, 51, 52, 53, 56, 58, 59, 66, 68, 73, and 82) are classified as carcinogenic to humans and cause anogenital and oropharyngeal cancers [23]. While the majority of people recover from an HPV infection within one to two years, 10% to 20% of infected women will continue to have the infection. Cervical cancers and a sizable portion of anogenital and head and neck cancers have been linked to chronic HPV infection, particularly with HPV types 16 and 18 [24]. HPV16/18 and HPV6/11/16/18/31/33/45/52/58 are carcinogenic and attributed to cancers of the cervix, other anogenital tracts, and head and neck. The burden of HPV-attributable cancers can be reduced by enhancing the programs for HPV vaccination and HPV-based cervical screening [25].

The age distribution of HPV infection indicates an early peak in the teens and twenties, followed by a gradual decline; however, in some nations, a second peak appears later in life [26]. Most cancers related to HPV affect women; consequently, sex-stratified estimates were considered. The global ASIR (age-standardized incidence) rate of cervical cancer is 13.1, with a mortality rate of 6.9 per 100,000 [27]. Cervical cancer is the second most common cancer in women under the age of 50 years, and the fourth most common cancer in women of any age.

In men, the global prevalence rate of genital HPV infection is comparable to that in women (3.5–45% vs. 2–44%) [28]; transmission rates are also similar [29]. Homosexuals and HIV-infected men are at higher risk, with higher rates of HPV anal infection (≥90%) compared with heterosexual men, whose risk of HPV infection is determined by the number of sexual partners [30,31]. This tendency differed from that observed in women. In terms of regional distribution, the incidence of HPV infection in men is higher in Africa, particularly among South African men (17.2% per year), and lower in Asia (3.2% per year) [32]. Giuliano et al. showed that a higher prevalence of all HPV genotypes was found in low- and middle-income countries than in developed countries [33]. In 2020, India accounted for 24% of HPV-related cancer cases and 7% of all cancer cases worldwide [34]. Four of the five cervical cancers reported in India were caused by HPV types 16 and 18 [35]. Furthermore, as part of a cancer prevention strategy in India, health facilities are implementing opportunistic screening for common cancers such as oral and cervical cancer [36]. Population- and hospital-based cancer registries in India can be used to track the effects of primary and secondary preventive interventions. A thorough understanding of the epidemiology of cancers linked to HPV infection would help define a country’s preventive intervention strategies.

While studying the global impact of HPV from the GLOBOCAN 2018 data, the World Health Organization (WHO) has called for global action towards the elimination of cervical cancer (a threshold of 4 per 100,000 women years) and has set targets to be accomplished by 2030 [37,38,39]. Almost all sexually active individuals, regardless of their gender identity, sex, or sexual orientation, are infected with HPV during the first couple of years of sexual activity, and nearly half of them are infected with high-risk (HR) HPV types. HPV is responsible for almost all cervical cancers and plays a major role in other cancers, including anal (90%), vaginal (75%), oropharyngeal (70%), vulvar (69%), and pancreatic (63%) cancers. More than 50% of the morbidity and mortality of vaginal and penile cancer cases occur in Asia, and vulvar cancer is predominant in Europe [34]. As reported in several parts of the world, the three currently approved HPV vaccines, bivalent, tetravalent, and 9-valent, are potent in lowering HPV infection. They target and induce immunity against low-risk (LR) and HR HPVs, which are responsible for 70 and 90% of genital and cutaneous warts and cancers, respectively. Moreover, available (bivalent, tetravalent, and 9-valent) vaccines based on virus-like particles have been proven to be effective and safe [3]. Among these, the new 9-valent vaccine appears to be a favorable next-generation vaccine.

## 3. HPV Molecular Structure and Classification

**Figure 1:**
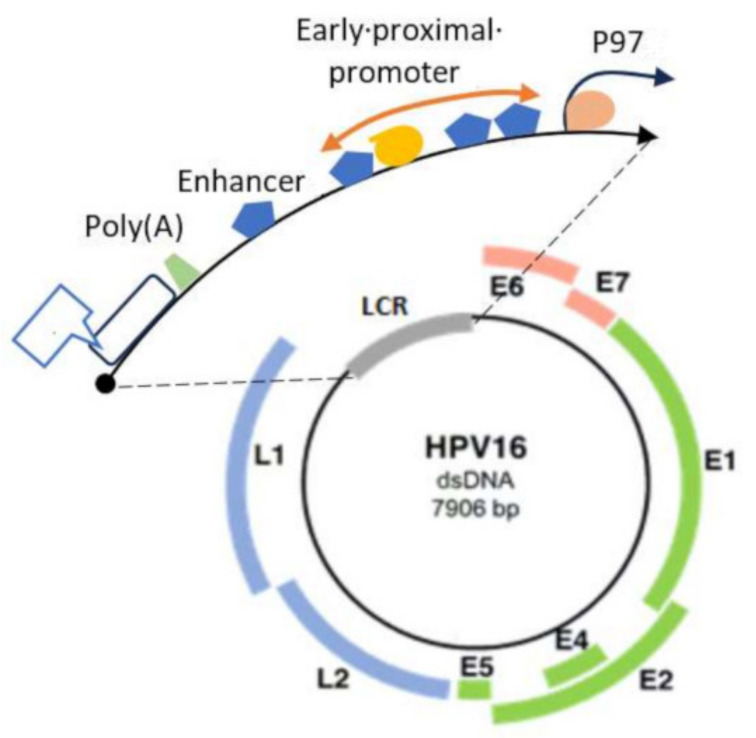
The structure of the HPV16 genome. The figure was modified and redrawn from [42].

HPV is a nonenveloped, covalently closed, circular DNA virus. It contains an ~8 kb genome and is divided into three main regions (Figure 1): early (E), late (L), and long Control Region (LCR) segments [40,41].

Early (E) region: The early region genes E1, E2, and E4–E7 constitute a major part of the HPV genome. E1, E2, and E4 are responsible for DNA replication, E2 also functions as a transcriptional repressor of E6/E7, E5 is responsible for cell transformation and proliferation, and E6 and E7 regulate the cell cycle. This early region is necessary for DNA replication, viral particle synthesis, discharge, and cell transformation [43].

Late (L) region: Approximately 40% of the HPV genome is composed of late-region genes, L1 and L2, which encode structural capsid proteins.

Long Control Region (LCR): This region is also known as the upstream regulatory protein (UPR) and forms 10% of the HPV genome. It is a noncoding segment with the origin of replication and transcription factor (TF)—binding sites that participate in viral gene transcription regulation to control DNA replication [44].

Depending on the HPV genome structure and its tropism to human epithelial tissues, there are more than 200 HPV genotypes, which are grouped into five different genera (alpha-, beta-, gamma-, Mu-, and Nu-) based on their life cycle and cause of infection [40,45,46]. Among them, the alpha genus is the largest group, and the HPV genotypes belonging to the alpha genus are responsible for cancer. Although HPVs under beta and gamma genus generally show asymptomatic effects, they can generate immunosuppressive stages, which can instigate other types of skin cancers or cutaneous papilloma and sometimes mold their hosts to complete the life cycle without generating any noticeable disease [43,47,48]. According to the risk levels of activation of oncogenic ability, HPVs are further classified into low-, intermediate-, and high-risk groups, and potentially generate infected cell proliferation and malignancy [49,50]. HPV6, 11, 42, 43, and 44 belong to the LR category and are responsible for condylomas and benign cervical lesions with no malignancy [50,51]. The intermediate oncogenic risk group contains HPV31, 33, 35, 51, and 52, which cause malignant transformation; however, this remains a controversial issue [23,50]. The high-risk (HR) group of HPVs includes HPV16, 18, 45, and 56, which are predominantly involved in triggering neoplastic transformations [23]. Furthermore, 15 HPVs belonging to the alpha group, including HPVs 16, 18, 31, 33, 35, 39, 45, 51, 52, 56, 58, 59, 68, 73, and 82, are classified as ‘HR’ types and have oncogenic properties and are responsible for causing anogenital cancers [42,52]. However, HPV 16 is responsible for nearly 55% of cervical cancer cases, while HPV 18 is responsible for 15% of cancer cases, and the remaining percentages of cancer cases are caused by other HR types [42].

## 4. HPV Infection and Its Role in Cancer Progression

HPV can be transmitted through sexual or nonsexual interactions. However, sexual transmission is the most common cause of HPV infection, especially the genital type [53]. Women, particularly those with several sexual partners, are most frequently affected by this infection. Sometimes, HPVs are transferred from mother to child through the perinatal transmission of other viruses or microbes [54,55]. In one report, HPV infection was horizontally transferred to a 5-year-old child through genital finger transmission via warts on his hands and anus [56].

**Figure 2:**
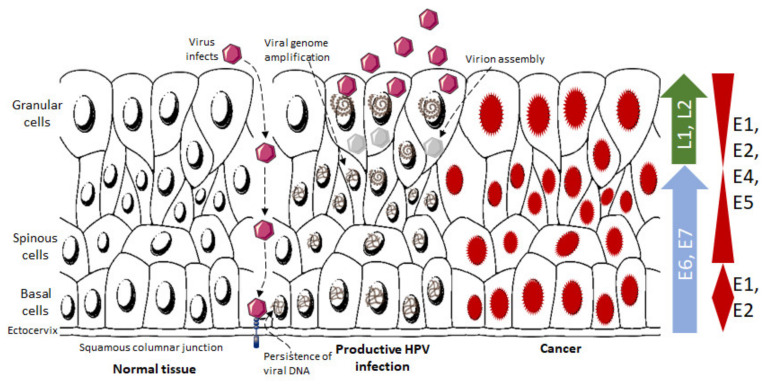
HPV infection in the cervix, viral particles shedding and HPV protein expression. The figure was modified and redrawn from [44].

First, Human Papilloma Virus enters the body through skin abrasions/warts, epidermal injuries, and mucous membranes (Figure 2) [53].

There are three main regions in the cervical mucosa: the endocervix, made up of simple columnar epithelium; the transformation zone, which contains both squamous and columnar cells; and the ectocervix, which forms the non-keratinized stratified squamous epithelium. Stratum basale, also known as stratum germinativum or the basal layer, acts as a parent layer for the formation of new cells [57]; therefore, a basal cell undergoes cell division via mitosis into two daughter cells: one grows and becomes differentiated, while the second cell is retained in the basal layer for further cell division. HPVs target basal cells and are transmitted to the epithelium via microabrasions/microwounds [58,59]. Furthermore, HR-HPVs enter the junction of the endo- and ectocervix through single-layer squamous cells [60]. For effective infection, these viruses attack actively dividing basal-layer cells, which act as stem cells [61,62]. Cellular receptors bind to the HPV L1 capsid protein [63]. Several kinds of cellular receptors are reported for HPV entry on the basis of HPV genotype, infected cell types, and various receptor attachment strategies, including integrins (α6 integrin) [59,64,65], epidermal growth factor receptors (EGFRs) [65], laminins [66], the annexin-A2 heterotetramer [67], vimentin [68], tetraspanin-enriched membrane microdomains [69] and syndecan-1 [65,70]. After initial binding of HPV to a primary receptor such as heparin sulphate proteoglycans (HSPGs) [71], there is a conformational change facilitated by cyclophilin B that occurs at the N-terminus of the L2 region, a viral capsid protein present on the virion surface [72], resulting in L2 cleavage by proprotein convertase (PC), furin, and/or PC5/6, followed by N-terminus L2 epitope exposure and binding with a secondary receptor present on the target cell plasma membrane [73,74]. HPV enters target cells via endocytosis in a manner similar to micropinocytosis [75]. Once the virus attaches to the cell, it enters the nucleus in nearly 24 h through post-endocytic trafficking via membrane-bound endosomes, the Golgi network, and the endoplasmic reticulum [76,77]. Ultimately, the viral genome is transferred to the nucleus via the tubulin-mediated pathway through nuclear pores or fragmented nuclear membranes in the mitosis process [78], which then associates with promyelocytic leukemia (PML) nuclear bodies to form a nuclear infection [79] and initiates viral transcription [80].

During microinjuries, HPVs generally infect the basal layer of the squamous epithelium. In basal or para-basal cells, E6 and E7 proteins are expressed to stimulate cell division in the epithelium [10,81]. During cell division, HPV DNA replication begins using cellular machinery. HPV DNA replication is initiated by E1 and E2 proteins, which are expressed in the middle epithelial cell layer. The E4 protein is expressed in the cells of the middle to upper epithelium, which breaks the keratin links of the host cell cytoplasm and pauses cell division. This protein enables the proliferation and release of HPV particles from the dying host cells. In these HPV particles, the HPV genome is inserted into the viral coat and assembles with L1 and L2 proteins in the uppermost epithelial layer. Most HPV proteins are expressed in the upper epithelial layer during productive infection; however, E6 and E7 proteins are expressed in the basal cell layers [10].

## 5. Molecular and Genetic Basis of HPV-Induced Carcinogenesis

When the virus enters the nucleus of basal layer cells, early transcription begins with the expression of E1 and E2 (viral replication/transcription factor) proteins, which are needed for replication, partitioning of newly formed DNA, and positioning the HPV genome into the cellular chromosome [59,82,83]. Early transcription of the viral transcription factor E2 controls the expression of regulatory proteins (E6 and E7) by regulating their promoter regions, and E6 and E7 proteins are necessary for the survival of HPV-infected cells [84]. The E2 protein contains two regions, one for DNA binding and the other for the protein-binding region, which prepares a homodimer for binding with four palindromic regions present on the LCR, also known as upstream regulatory region (URR), and its three regions are situated near the origin of replication [83]. E2 binds to viral helicase E1, which interacts with and efficiently recognizes the origin of replication, recruits the machinery for DNA replication, and initiates viral genome replication [42,85,86]. During replication, the HPV genome produces nearly 50–100 copies per nucleus. This controlled genome replication occurs in undifferentiated cells because of the expression of E8^E2, a highly conserved viral repressor protein composed of a combination of the C-terminal halves of E2 and E8 proteins [87]. It binds to specific DNA sequences and inhibits viral replication. The E8 protein has repressive properties through interactions with cellular NCoR/SMRT corepressor complexes [87]. The E8^E2-fused protein controls the replication process in undifferentiated cells and facilitates constructive replication in differentiated cells [87]. In infected basal cells, viral genome replication and equal division into daughter cells through association with host cellular chromosomes via E2 attachment to mitotic chromosomes are facilitated by contact with Brd4 (a human bromodomain protein) [88]. Brd4, like other proteins, has been reported to anchor the HPV genome to host chromosomes, including TopBP1 [89], ChlR1 [90] and MKlp2 [91]. The E2 protein also suppresses the P97 promoter by limiting the access transcription of promoter transcription factors as well as by changing chromatin conformation in infected cells to eliminate stimulation of the local immune response [92,93,94]. Through this process, HPV can persist in the epithelial cells for a long time. Infected basal cells then divide and develop transit cells with a viral genome that can move towards the upper epithelial layer [95]. Viral gene replication and expression are conducted with the process of epithelial differentiation. HPV16 and HPV18 have early promoters, such as P97 and P105, respectively, which are responsible for the protein expression of early-stage replication cycles such as oncoproteins E6 and E7 [96]. The E6 protein is required for the maintenance of the episomal genome, and the E7 protein is responsible for the activation of the G1 to S-phase checkpoint and potentially controls transcriptional alterations in infected cells [97,98,99]. E7 activates the cell cycle in differentiating infected cells through interactions with pRb (retinoblastoma tumor suppressor protein) and other proteins p107 and p130 (called pocket proteins) via the sequence LXCXE in CR2 sequences [100]. These pocket proteins control the function of E2F family transcription factors, which regulate multiple cell cycle transitions. E2F releases and stimulates the expression of cell cycle-associated genes, cyclins A and E, which activate the conversion of the cell phase from G1 to S [84,101,102]. The E7 protein can also directly interact with E2F1 in a retinoblastoma-independent manner in an in vitro and in vivo study, in which the E7 protein generated by the HR-HPVs binds more tightly to E2F1 than to LR-HPVs and activates E2F1-dependent transcription and promoter activities, which participate in the deregulation of the cell cycle and induction of transformation [103]. Some studies have reported that the expression levels of E6 and E7 increased in the lower and middle-upper layers of the epithelium [104,105] but decreased in the epithelial upper layer [51]. E7 is always present with E6 during the viral life cycle because of the bicistronic nature of E6/E7 genes in the viral genome [106]. After viral integration, the expression of the E6/E7 genes was constantly maintained, whereas other genes were deleted or not expressed properly [107]. Two main HPV transcripts are generated: one encodes both E6 and E7 proteins, and the second encodes the complete part of the E7 protein and the spliced E6 protein [108]. Most HPV transcripts are spliced using cellular splicing and the polyadenylation processes and may enhance the stability of HPV transcripts in cervical cancer cells [108].

### 5.1. E7 Protein

**Figure 3:**
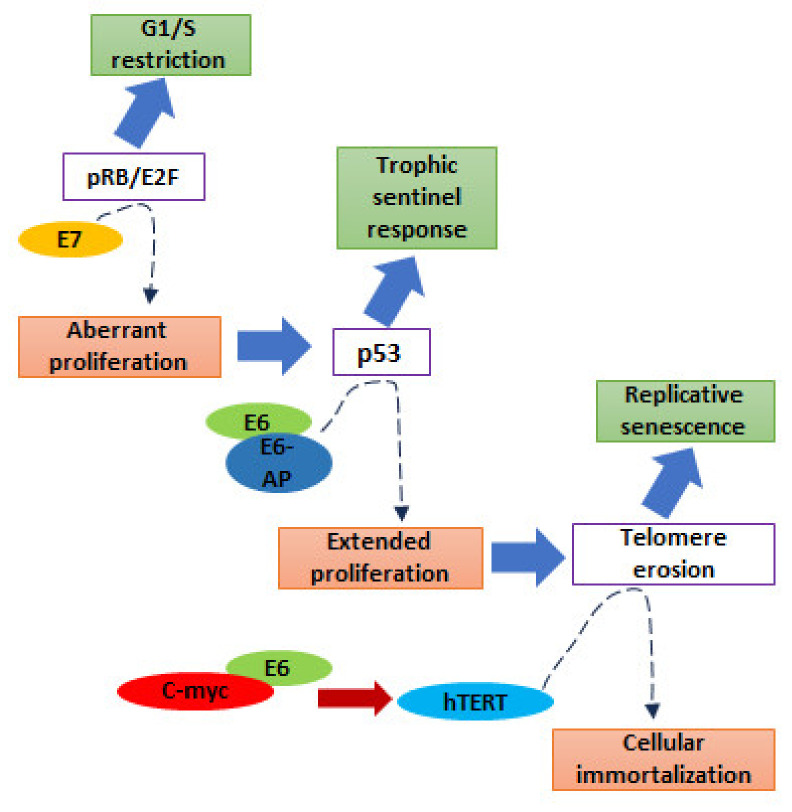
The figure presented a schematic representation of HR-HPV-inducing carcinogenesis. The figure was redrawn from [108].

E7 acts as a regulator of the G1/S checkpoint (Figure 3). It induces neoplastic activity through its interaction with and further inactivation of retinoblastoma protein (pRb), whose phosphorylation state depends on the cell cycle stage; that is, in the G0 and G1 phases, it is dephosphorylated. Phosphorylation inactivates the pRb, which in turn triggers uncontrolled cell proliferation. The pRb regulates the cell cycle by interaction with the E2F transcription factor in a phosphorylation-dependent manner. The Rb protein is phosphorylated by cyclin D kinases (CDKs) in the G1 phase, whereas after phosphorylation, it cannot interact with E2F, and its inhibitory effect is no longer observed; thus, the cell enters the S phase [44,109]. During the S phase, the Rb protein remains phosphorylated until the hypophosphorylated state cannot be developed by a specific phosphatase in later stages of the M phase [110]. These activities are essential for the replication and life cycle of HPV [111]. In contrast, when damaged DNA is detected, p53 is activated, further stimulating p21 (a CDK inhibitor). The p21 restricts the function of cyclin E-CDK2, which does not phosphorylate the pRb. Therefore, the Rb protein can bind to E2F and inhibit the function of E2F, thereby stopping the G1 to S transition [44].

The E7 oncoprotein also controls DNA methylation by regulating cell propagation pathways; thus, epigenetic alterations occur via the Rb family of tumor-suppressor proteins [112]. HPV-16 E7 has been shown to bind to DNA methyltransferase DNMT1 in vivo as well as in vitro studies for its activation [113]. When E7 interacts with Rb and E2F is removed from the inhibitory complex, E2F binds to DNMT1, which is responsible for the hypermethylation of CpG islands [114].

### 5.2. E6 Protein

In HPV-infected cells, the E6 protein primarily represents the neoplastic effect abrogation of p53 depending on ubiquitin (Figure 3) [59].

p53 is a tumor-suppressor gene and a key regulator of apoptosis that stops the generation of cancer-causing destructive mutations. These mutations can cause DNA damage and errors during replication. In such a situation, in the presence of abnormal DNA, p53 activates, the cell cycle ceases, and the DNA repair mechanism begins before cell separation. Otherwise, cells undergo apoptosis when the DNA is not repaired [111]. In cervical cancer cells, the level of p53 is lower than that in healthy cells, and its half-life is short. Therefore, mutations persist in an unrepaired form and are carried forward to the next generation, ultimately leading to genomic aberrations [110]. These conditions are deficient in DNA repair mechanisms and encourage the mutagenesis of cancer cells.

The binding of E6 to p53 depends on E6-associated protein (E6AP). E6AP is an E3 ubiquitin protein ligase that performs proteasomal degradation via substrate recognition through ubiquitination machinery [110]. HR E6 proteins inhibit the transcriptional activity of p53 by binding to the histone acetyltransferase CBP/p300 [115,116] and induce conformational alterations in p53 [117]. E6 also accomplishes gene silencing through DNA hypermethylation by increasing the DNMT1 levels. Degradation of p53 liberates the specificity protein 1 (Sp1) transcription activator, which interacts with the DNMT1 promoter and upregulates its expression [112].

## 6. HPV Infection Generates Host Immune Response

**Figure 4:**
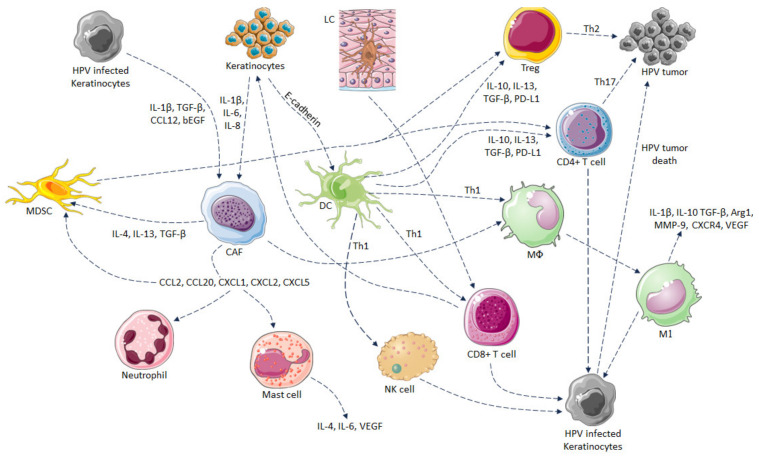
HPV-related carcinogenesis hypothesis linking stromal cells, such as cancer-associated fibroblasts (CAF), myeloid-derived suppressor cells (MDSCs), etc., immune cells and keratinocytes. The figure was modified and redrawn from [136].

Because the host immune response can combat HPV, specific symptoms do not often occur during HPV infection. However, the recurrence of HPV infection develops in special situations, including in people with immune disorders, sexual partners, comorbidities, and elderly persons. Particularly during infection with HPV16 and HPV18, viruses cause cancer progression in a few cases [118]. Both the innate and adaptive immune responses are generated to combat HPV infections. During infection or sexual contact, highly specific antigen-presenting cells (APCs), such as Langerhans cells (LCs), are activated and represent HPV proteins on their surfaces. LC levels were significantly lower in the transformation zone than in the exocervix. In addition, an increase in the number of LCs was observed in squamous intraepithelial lesions (SILs), although they showed inadequate action [9,119]. In addition, during HPV16 L1 infection, LCs are unsuccessful in generating an appropriate immune response owing to their immune tolerance [10]. Localization of LCs is expected to be reduced in the epidermis because of lower E-cadherin expression in the epidermis caused by most HPV species [120]. In epidermis that do not have lesions, LCs are unable to induce a satisfactory immune response compared to DCs of the dermis because of the absence of proper costimulatory signals [121]. In the skin, DCs and monocyte-macrophages play a major role in recognizing HPV antigens and utilize major histocompatibility complex (MHC) molecules to represent the antigens. These cells are immunologically induced by interactions with various viral elements, including ssDNA, viral RNA, CpG motifs, and Toll-like receptors (TLRs), and represent antigenic epitopes on their surface [10]. Effective innate immune responses are generated via TLRs, which recognize specific patterns of microbial components, such as PAMPs/DAMPs, and stimulate the production of type 1 interferons (IFN), defensins, and proinflammatory cytokines, such as TNF-α, IL-1β, IL-6, and IL-12, which induce local inflammation [9,122,123,124]. These signals are required to induce an adaptive immune response. TLR 9 significantly distinguishes the ds DNA of HPV virus and starts the cascade process of INF-α, INF-β, and INF-γ interferons [125]. However, the HPV oncoproteins target both TLRs and interferon pathways, resulting in an irregular expression pattern that enhances viral persistence as well as the carcinogenic process [126]. The oncoproteins E6 and E7 of HPV16 lowered the expression of TLR9 [127]. In addition, INF-γ and IL10 expression were interrupted in the malignant epithelium by methylation of the promoter region or by oncogenes [128,129]. Cytokine secretion activates the macrophages, which can destroy HPV-affected cells through the production of TNF-α or antibody-dependent cytotoxicity [130]. Monocyte chemotactic protein-1 (MCP-1) chemokine is secreted by keratinocytes (KCs) under the influence of TNF-α, which can attract macrophages at the site of infection, however, its secretion is reduced by the E6 protein of HPV 16 [131]. Similarly, E6 and E7 proteins of HPV 16 influence the expression of another monocyte chemoattractant chemokine Macrophage inflammatory protein (MIP)-3α [132]. NK cells also play an important role in innate immune response to fight against viral infected cells or tumor cells that do not present MHC molecules on their surface. NK cell deficiency was found in cervical cancer patients due to downregulation of NK cell receptors expression, including NKp30, NKp44, NKp46 and NKG2D on the surface of NK cells, resulting in lowered NK cell cytotoxic activity against tumor cells [133]. Likewise, in the patients of severe combined immune deficiency (SCID) who received hematopoietic stem cell transplantation therapy, which is a life-saving treatment, those having a deficiency in NK cells or gamma(c)/JAK-3-dependent signaling represented inadequacy in the generation of immune response against HPV infection [134]. When CD1d interacts with CD1d-restricted natural killer T (NKT) cells, it activates NKT cells, releases cytokines and stimulates adaptive immune responses, and acts as a link between the innate and adaptive immune responses, whereas during infection, HPV6 or 16 expression of CD1d is downregulated by the influence of E5 protein by the immune escape mechanism [135]. Figure 4 indicates the host–microbe interplay in HPV-induced invasive cancer.

Innate immunity plays a key role in the primary removal of viral infection, and adaptive immunity is responsible for the retrogression of lesions. The HPV oncoproteins E2 and E6 are recognized by TH1 (CD4+ T helper 1) cells that secrete IL-2, IFN-γ, and CD8+ Tc cells and facilitate the clearance of low-grade HPV infection. However, high-grade neoplasia can be regulated by distinguishing E7 proteins from CD4+ TH1 cells [10,137,138]. In addition, CD8+ cytotoxic T cells are considered imperative factors for the exclusion of HPV infection and increasing patient survivability [139,140]. During HPV infection, specialized APCs process HPV proteins into antigenic peptides expressed on MHC II molecules on their cell surfaces, which recognize CD4+ helper T lymphocytes and initiate an adaptive immune response. The generated immune cells secrete different cytokines, such as interleukin (IL)-1, IL-6, TNF-α, and IL-12, which direct the immune response, generate local inflammation, and act as danger signals [9,10]. After activation, CD4+ Th cells differentiate into Th1 and Th2 cells. Th1 cells stimulate cell-mediated immunity, while Th2 cells activate antibody production [10]. Th1 cells release cytokines, including IL-2, IL12, and IFN-γ, which activate differentiation of the CD8+ T cells into cytotoxic T lymphocytes (CTLs). These CTLs act as effector T cells that kill cancer or CIN cells, representing HPV antigens. CIN is a type of cancer-stage dysplasia that occurs in cervical squamous cells and acts as a potent premalignant transformation primarily generated by HR-HPV 16 and 18 [141]. CIN is grouped into three types: (1) CIN1 (mild dysplasia), (2) CIN2 (moderate dysplasia), and (3) CIN3 (severe dysplasia, and carcinoma in situ) [141]. CTLs secrete granzyme B and perforins and perform histological regression in the CIN stages. Immunohistochemical studies have reported that CD8+ T cells present in CIN1 and koilocytes in cervical lesions expressed α4/β7 integrin [124].

## 7. Immune Escape Mechanism for HPV Perseverance

HPV uses a variety of immune evasion mechanisms to suppress immune responses and promote cancer progression [142]. HPV can show an immune escape strategy not only by hiding itself from recognition by immune cells by downregulating viral antigens, but also by disturbing the expression of immune response proteins, which further encourages prolonged viral persistence [142]. Another mechanism of immune escape involves viral particles formed by the natural process of cell shedding in the uppermost epithelial layer, where limited access to the immune cells facilitates HPV evasion from immune recognition and the immune response [143]. In addition, HPV disturbs the DNA methylation status and modifies the gene expression of host cell proteins, especially by downregulating significant immunomodulators, such as cytokines/chemokines, adhesive molecules, and TLRs [144,145]. HPV also hinders protein–protein interactions in host cells by interrupting protein function [145]. HPV16 alters the expression of proteins involved in antigen processing, such as the immunoproteasome subunits PSMB8 and PSMB9, in infected cells [146]. E5 oncoprotein of HPV16 lowers the cell surface expression of HLA class-1 molecules on the APCs by retaining them into the Golgi compartment, which could interfere in the action of the CTL attack [147,148]. The E5, E6, and E7 proteins of HPV16 interrupt the interferon pathway by interacting with transcription factors (interferon response factors) that activate interferon genes [149]. HPV18 dysregulates the expression of the cyclic guanosine monophosphate–adenosine monophosphate synthase-stimulator of interferon genes (cGAS-STING), which represents a defense strategy against DNA viruses [150]. In an in vitro study, HR-HPV (HPV16 and HPV18) episomes transfected to KCs illustrate the disturbance in of inflammatory cytokine/chemokine expression [123]. HR-HPV expressing E6 and E7 decreases the expression of TLR9 and hinders its function in viral particle recognition [151,152]. These E6/E7 oncoproteins disrupt NFκB signaling by obstructing NFκB translocation to the nucleus via hindrance of its transcriptional activities and inhibition of its production of TLR-mediated proinflammatory cytokines/chemokines, which prevent trafficking of innate immune cells and stimulation of appropriate Ag-specific effector cells [153,154]. Further KCs infected with HPVs do not generate type-I IFN and proinflammatory cytokines, including IL-6, IL-8, TNF-α, and MIP3a [153,154]. HR-HPV infection also stops the TNFα-associated necroptosis and IFN-γ-associated cell-cycle arrest via decreased expression of receptor-interacting protein kinase 3 (RIPK3) and interferon-induced transmembrane protein 1 (IFITM1), respectively [155]. HPV can acquire all these strategies to evade an effective immune response, which may eventually support the prolonged persistence of HPV infection and cervical cancer progression.

## 8. Prevention Strategies to Control HPV-Associated Cancers

There are three major strategies to regulate HPV infection as well as mortality and incidence rate by its infection or by its consequent cervical cancer: (1) screening (pap screening) for early detection of cancer, (2) vaccination programs, and (3) treatment coverage, which are considered the most effective preventive strategies [156]. These three core activities can be efficiently monitored by measuring the incidence of natural history of HPV infection, cervical lesions, and cancers to reduce their occurrence. Comprehensive surveillance programs at national or subnational levels targeting these three pillars (screening, vaccination, and treatment) are a framework in the WHO Global Strategy to reduce cervical cancer for public health concerns [157]. The aim of the strategy is to achieve targets by 2030, especially in low- and low-middle-income countries. This involves screening 70% of women at the age of 35 and repeating the screening at the age of 45, ensuring that 90% of girls are fully vaccinated by age 15 with the HPV vaccine, and ensuring that 90% of women diagnosed with cervical disease receive proper treatment.

- (A) Screening:

The early detection of HPV plays a critical role in the prevention and management of HPV infection and cervical cancer progression. Regular screening is critical for monitoring disease progression or regression as HPV infection is present in a latent form. HPV infections (DNA or mRNA) in cervical or vaginal samples can be detected using the HPV nucleic acid amplification test (NAAT) [158,159]. The primary test to detect HPV infection is a nucleic acid base, while the examination of exfoliated cells by microscope to detect the HPV-induced changes in the cervical epithelium is known as the Papanicolaou (Pap) cytology test (Pap test or Pap smear) [160]. These tests illustrate that HR-HPV infections and abnormal cell changes, such as precancerous alterations, can be treated earlier before they are converted into cancerous cells. In addition to HPV NAATs and cervical cytology, visual inspection with acetic acid (VIA) can also be used [161]. Therefore, VIA screening is useful in locations with limited medical facilities. In this test, only a few tools are required, and a dilution of white vinegar is applied to the cervical region, which turns white upon contact with the vinegar; any abnormalities can be observed by the health care server (cancer.net). The WHO, American College of Obstetricians and Gynecologists (ACOG), and American Cancer Society (ACS) recommend the Pap smear test every three years for women aged 21–65 and more frequently for immunocompromised women [162,163]. Nucleic acid-based tests are available for the early detection and/or screening of HPV-infected cervical cancer; however, there is no parallel system for screening HPV infection in other cancers. Further research is required to develop methods for the detection and/or screening of HPV infection in other malignancies, such as oropharyngeal cancer [164]. Surveillance-based programs for effective cervical screening should be scaled up and strengthened to support the elimination strategy using survey-based methods, population-based screening studies, encouraging women to participate in screening programs and regular screening based on cytology or VIA, and extending the infrastructure and healthcare staff facilities in resource-limited areas [156].

- (B) Vaccination:

The HPV vaccination program against HPV was started in 2006, and Austria was the first country to introduce a government-funded National Human Papillomavirus (HPV) Vaccination Program. Before the recommendation of the World Health Organization in 2009, most high-income countries had already started funding national HPV vaccination programs [165,166]. To date, 131 countries (67.52% of member states) have fully adopted HPV vaccination and three have partially adopted HPV vaccination in their national immunization schedules. However, the world’s most populous countries, that is, India, China, Russia, and most Central and Middle East Asian and African countries, have not started nationalized immunization.

Currently, six HPV vaccines are available: three bivalent (cervarix, cecolin, and walrinvax) that are effective against HPV-16 and 18; two quadrivalent (gardasil and cervavax) that are effective against four strains (HPV-16, 18, 6, and 11); and one nonavalent (gardasil 9) that is effective against the maximum number of strains (HPV-16, 18, 6, 11, 31, 33, 45, 52, and 58). These vaccines are based on virus-like particles (VLPs) that self-assemble in the L1 capsid protein when expressed by recombinant DNA vectors and provide immunogens for prophylactic vaccines [167,168].

Nearly 134 countries have HPV vaccination programs and mostly target females only [18]. High-income countries have better vaccination coverage than other countries. To date, of more than 200 HPV genotypes, only 14 have been classified as carcinogenic (HR HPV) in humans by the International Agency for Research on Cancer (IARC), namely HPV genotypes 16, 18, 31, 33, 35, 39, 45, 51, 52, 56, 58, 59, 66, and 68 [169]. Among various carcinogenic HPV types, only two, namely HPV16 and HPV18, are highly potent carcinogens and are responsible for the majority of the disease burden [170]. The mathematical model and meta-analysis recommended that vaccination will lead to a strong herd effect, and nearly 80% of HPV vaccinations in both sexes could eliminate HPV-16, 18, 6, and 11 [171]. Recent studies in the United States, Austria, Canada, Switzerland, and Australia observed that targeting both sexes, i.e., gender-neutral vaccination (GNV), compared to female only vaccine, has the potential to decrease HPV-associated disease significantly [171]. In 2022, the Strategic Advisory Group of Experts on Immunization (SAGE) of the World Health Organization (WHO) concluded, on the basis of evidence over the past years, that single-dose vaccines provide strong efficacy against HPV comparable to 2–3 doses. This recommendation has a tremendous effect on the vaccination and prognosis of HPV in LMICs, as more life-saving jabs are available [172]. However, the data are opaque regarding individuals who have compromised immune systems, including those with HIV, and SAGE suggested that they should receive at least two doses and, if feasible, three doses [172].

The WHO recommended a national immunization schedule for preliminary young girls aged 9 to 14 years before they become sexually active, which helps to significantly prevent HPV infection and associated cancer mortality; secondary targets are women over 15 years of age [173]. Research has shown that HPV vaccines are safe and facilitate the reduction of HPV infections, genital warts, and HPV-associated cancer [174,175,176,177,178]. Although HPV vaccination has several benefits, the global coverage of immunization is far from the target, and at least 80% coverage is required to eliminate HPV infection [179]. To achieve the elimination of cervical cancer in this century, the WHO targets fully vaccinating 90% of girls at the age of 15 years, screening 70% of women 35–45 years, and treating 90% of women who have cervical cancer by 2030 [180]. To eradicate cervical cancer, a successful model adopted by high-income countries for immunization will provide a roadmap for LMICs. The successful model adopted by high-income countries for immunization against HPV infection will provide a roadmap for LMICs to eradicate cervical cancer. One of the most successful pioneers is the Australian national HPV immunization program, which had over 89% and 86% coverage in girls and boys at age 15, respectively, for the single dose in 2017 and had among the highest coverage rates in the world [181]. In 2007, Australia started a highly efficient government-funded school-based delivery of three doses of a quadrivalent vaccine, which also involved multi-age cohorts of women (18–26 years of age). It is predicted that cervical cancer will be reduced to less than four cases per 100,000 women per year by 2028 and will have a very high probability of becoming the first nation to eliminate cervical cancer by 2040 [182,183]. With a vaccination coverage of 90%, the Flanders region of Belgium is another success story in the European Union; vaccine awareness among health providers, better school health services with an improved vaccination database, less vaccine hesitancy, and the influence of advertisements are successful pioneers in this community compared to other regions [184]. In Sweden, since 2010, the introduction of a fully funded government school-based program of quadrivalent vaccine covers more than 80% of the population; this mode of the program has reduced social disparities and showed higher efficiency in immunization [185,186]. A recent survey with a 93% positive attitude toward vaccines in Sweden is another example where vaccine hesitancy in lower-educated and socioeconomic communities was changed toward positive vaccination by well-aware health providers [185].

- (C) Treatment:

An effective treatment strategy involving screening surveillance programs is crucial to reduce the morbidity and mortality of cervical cancer. The WHO Health Organization elimination strategy aims to achieve these goals by scaling up effective treatment rates to maximize optimal care facilities or palliative care services, especially in low-resource settings, to decrease cervical cancer incidences [187,188]. Surveillance of treatment approaches is critically important to prepare a bridge between public health screening programs and clinical services for the timely management of the disease. The appropriate treatment for cervical cancer depends on specific settings such as healthcare facilities, resources, services, and human resources [189]. Transformation of data and information between local healthcare centers (where screening facilities and primary care services are available) and cancer centers (where optimal care facilities are present and deciding which type of therapies such as surgery, radiotherapy, chemotherapy, and other palliative care refer to the patients) is critically important [156]. Additionally, electronic healthcare datasets with subsequent healthcare records (e.g., hospitalization, emergency, and cancer treatment service records) should be properly maintained in pathology and cancer registry records [156]. Various treatment methods for HPV-associated infections include surgical excision, vaginectomy, cryotherapy, electrocautery, loop electrosurgical excision, carbon dioxide (CO_2_) laser, salicylic acid, Imiquimod, Trichloroacetic acid, podofilox, and Brachytherapy, among others [190]. However, there is no consensus on the best method. Management and diagnosis of vaginal intraepithelial neoplasia (VaIN) are difficult; otherwise, it can evolve into invasive cancer. Treatment of the VaIN depends on the grade of the lesion, VaIN 1 (low-grade vaginal squamous intraepithelial lesions (SIL)) can be followed up, whereas VaIN 2–3 (high-grade vaginal SIL) should be treated [190]. VaIN 1 shows a high potential for regression in 48.8–88% of cases without treatment and has a low risk of progression. Clinical data suggested that observation illustrated better results in comparison to recurrence, which occurred in 22–24% of cases after treatment with a laser or excursion [191,192,193,194]. High-grade lesions of the vagina (VaIN 2/3) have a 9–50% probability of progressing to invasive cancer without treatment [195]. The treatment choice depends on the characteristics of the disease (extent of disease, severity, and site of disease), patient background and history (age, immune status, parity, and sexual activities), prior treatment procedures, etc. [190]. The International Society for the Study of Vulvovaginal Disease (ISSVD), the European Society of Gynecological Oncology (ESGO), the European College for the Study of Vulval Disease (ECSVD), and the European Federation for Colposcopy (EFC) collaborated on consensus statements for the management of pre-invasive vulvar lesions. This initiative aims to improve the quality of care for patients infected with VaIN [190].

## 9. Vaccination Stratagems to Fight against HPV Infection

The Centers for Disease Control and Prevention (CDC) defines a vaccine as a preparation used to stimulate the body’s immune response against diseases. It trains our immune system to protect our body when exposed to a disease; it contains a weak or killed form of bacteria, peptides, nucleic acids, or proteins that do not cause disease or risk of complications. Although prophylactic HPV vaccines developed over the last decade have been extremely successful in preventing HPV infections, they are incapable of eliminating or treating HPV or HPV-associated lesions or infections [196]. HPV etiologic factors play a critical role in HPV-associated diseases such as cervical cancer, anal cancer, and non-cervical malignancies, and a cost-effective public HPV vaccination strategy will play a major role in eradication, even in places with low cervical cancer screening, especially among girls. Ideal HPV vaccines should have the following characteristics: safety, targeting cancer specificity, long-lasting effect, ability to potentially activate an HPV-specific immune response, minimum dose requirement, and last but not least, minimal cost [168].

Bacteria and viruses are utilized as delivery vehicles in live vector vaccination, to immunize the system; they infect the host, replicate in the body, and spread the antigen [197]. Live vector-based vaccines are immunogenic and are capable of boosting robust humoral and cell-mediated immune responses. Therapeutic HPV vaccines can deliver E6 and/or E7 antigens through live vectors to antigenic-presenting cells, and present antigens on cells via the MHC-I and MHC-II pathways [198].

Several bacterial vectors have been reconstituted for the delivery of antigens of interest to APCs and have been developed into therapeutic HPV vaccines. Some of these attenuated bacterial vectors include *Listeria*, *Shigella*, *Salmonella* and *Escherichia coli*. [199,200,201]. *Listeria monocytogenes*, a Gram-positive intracellular facultative bacterium, is a promising vector candidate for antigen delivery [202]. Live *Listeria monocytogenes*-based fused (pore-forming toxin, listeriolysin O) vaccine antigens are processed and presented through the MHC-I and MCH-II pathways, which can induce antigen-specific CD8+ and CD4+ T cell responses [203,204,205]. In preclinical trials, *Listeria monocytogenes*-based HPV E7 vaccine has been able to restrain the growth and burden of tumors in wild-type and transgenic mice by stimulating E7-specific CD8+ T cell response [206,207,208,209,210,211]. The immunogenicity demonstrated by *Listeria monocytogenes*-based HPV E7 vaccine in preclinical studies has been further translated into clinical studies. ADXS11-001, a fusion of a modified LLO molecule and HPV16 E7 protein, is a live, attenuated *Listeria monocytogenes*-based vector vaccine that has demonstrated safety and efficacy in women with cervical cancer (NCT01266460) and HPV-associated head and neck (NCT02002182) cancer in a clinical trial [201,204,212].

In addition to bacterial vectors, several live virus vector-based HPV vaccines have been explored for the development of therapeutic HPV vectors in preclinical and clinical trials owing to their high immunogenicity. To deliver HPV E2, E6 and E6 antigens, several live viral vectors, such as alphaviruses, lentiviruses, adenoviruses, adeno-associated viruses, and vaccinia viruses, have been used [201,203,213]. Among these viral vectors, the vaccinia virus, an envelope, and a double-stranded DNA virus have demonstrated high immunogenicity in clinical studies. In phase I/II clinical trials, live recombinant vaccinia-based vaccine expressing HPV16 and 18, E6/E7 proteins (TA-HPV) showed immunotherapeutic effects against cervical cancer [214]. Another modified vaccinia Ankara virus (MVA), encoding the bovine papillomavirus type 1 (BPV-1) E2 protein, showed a robust antigen-specific immune response and complete regression of precancerous lesions in patients with CIN1/2/3 lesions [215,216,217] and in a phase III clinical trial, it was also used for the treatment of HPV-associated intraepithelial lesions in anogenital [218]. Although live vector-based therapeutic vaccines have shown promising results in clinical trials, they have safety issues, particularly in immunocompromised individuals.

A long or short peptide derived from the epitope of the HPV antigen that is exposed to DCs for processing and presentation to MHC-I molecules can activate antigen-specific immune responses. Compared to live vector-based vaccines, peptide vaccines provide several advantages, such as stability, safety, and ease of production, but have weak immunogenicity and MHC restriction [201,219]. Adjuvants such as lipids, toll-like receptor (TLR) ligands, chemokines, and cytokines are used to improve the strength and efficacy of peptide-based HPV vaccines. In addition, some specific adjuvants that have been previously applied to improve the potency of HPV vaccines are the I*g*G fragment [220], aluminum adjuvants [221], the cytokine bryostatin that stimulates DC [222], and TLR agonists [221,223,224,225,226]. Another drawback of HPV peptide-based vaccines is that they are only effective against specific epitopes of the HPV antigen and require the identification of each individual. HPV peptide-based vaccines are major histocompatibility complex (MHC)-specific, which is a major challenge for the treatment of HPV-associated diseases and large-scale production. This challenge can be overcome by producing overlapping long-peptide vaccines that can stimulate antigen-specific T cells [227,228].

In a phase II trial, it was observed that two doses of the HPV16-SLP vaccine were capable of eliciting a robust and stout response in HPV16-specific T cells with low-level abnormalities in cervical cancer, and when combined with standard chemotherapy, they demonstrated enhanced immunogenicity in advanced cervical cancer [226,229]. This result led to the design of additional clinical Phase I/II studies to evaluate the potential of vaccines for advanced cervical cancer ((NCT02128126) and other HPV-associated malignancies (NCT01923116) [230]. There are several other studies in different clinical phases to evaluate peptide-based therapeutic HPV vaccines with different adjuvants also in trials, such as the PepCan ((NCT02481414) vaccine (four cGMP-manufactured synthetic peptides) in high-grade squamous intraepithelial lesions [231], with adjuvant Montanide™ and granulocyte-macrophage colony-stimulating factor with recurrent/metastatic squamous cell carcinoma of the head and neck [232].

## 10. Concluding Remarks

Human papillomavirus (HPV) is the most common sexually transmitted agent that causes lethal cancers, particularly cervical cancers. The high mortality and prevalence rates of cervical cancer, particularly in developing countries, have reached an alarming level and drawn urgent attention. The immunological components of the effective innate and adaptive immune responses are essential r for fighting viruses in the HPV lesion microenvironment. These immune responses have been endorsed using effective and safe vaccines that can stimulate the cytokine milieu, Treg cell generation, and cell-mediated immune responses for the clearance of HPV infections. HPV displays an immune evasion mechanism to escape the host’s immune response, particularly HR-HPVs, HPV-16 and -18. The 9-valent vaccine seems to provide satisfactory and effective results, but there is a need to develop broad-spectrum, next-generation vaccines that efficiently target HR-HPV variants and other HPV-existential proteins. Surveillance and awareness programs for diagnostics (screening), immunization (vaccination), and treatment should be budget- and resource-strengthened to reduce the burden of infection on women and prevent infection progression. Prevention of HPV infection is better than the treatment of cervical cancer; therefore, a nationwide vaccination program for HPV infection would help lower the prevalence of cervical cancer in the future if most girls and women participate in immunization programs.

## Data Availability

Not applicable.
